# RNA splicing factor RBFOX2 is a key factor in the progression of cancer and cardiomyopathy

**DOI:** 10.1002/ctm2.1788

**Published:** 2024-09-07

**Authors:** Jinze Shen, Jianqiao Shentu, Chenming Zhong, Qiankai Huang, Shiwei Duan

**Affiliations:** ^1^ Key Laboratory of Novel Targets and Drug Study for Neural Repair of Zhejiang Province School of Medicine Hangzhou City University Hangzhou China; ^2^ Medical Genetics Center, School of Medicine Ningbo University Ningbo China

**Keywords:** alternative splicing, pre‐mRNA, RBFOX2, therapeutic targeting

## Abstract

**Background:**

Alternative splicing of pre‐mRNA is a fundamental regulatory process in multicellular eukaryotes, significantly contributing to the diversification of the human proteome. RNA‐binding fox‐1 homologue 2 (RBFOX2), a member of the evolutionarily conserved RBFOX family, has emerged as a critical splicing regulator, playing a pivotal role in the alternative splicing of pre‐mRNA. This review provides a comprehensive analysis of RBFOX2, elucidating its splicing activity through direct and indirect binding mechanisms. RBFOX2 exerts substantial influence over the alternative splicing of numerous transcripts, thereby shaping essential cellular processes such as differentiation and development.

**Main body of the abstract:**

Dysregulation of RBFOX2‐mediated alternative splicing has been closely linked to a spectrum of cardiovascular diseases and malignant tumours, underscoring its potential as a therapeutic target. Despite significant progress, current research faces notable challenges. The complete structural characterisation of RBFOX2 remains elusive, limiting in‐depth exploration beyond its RNA‐recognition motif. Furthermore, the scarcity of studies focusing on RBFOX2‐targeting drugs poses a hindrance to translating research findings into clinical applications.

**Conclusion:**

This review critically assesses the existing body of knowledge on RBFOX2, highlighting research gaps and limitations. By delineating these areas, this analysis not only serves as a foundational reference for future studies but also provides strategic insights for bridging these gaps. Addressing these challenges will be instrumental in unlocking the full therapeutic potential of RBFOX2, paving the way for innovative and effective treatments in various diseases.

## Graphical abstract

RNA‐binding fox‐1 homologue 2 (RBFOX2) binds to RNA, influencing splicing and recruiting PRC2 for transcriptional repression. RBFOX2 impacts electromyographic signalling, L‐type Ca^2+^ channels, sliding filaments and mitochondria. RBFOX2 is involved in tumour cell proliferation, invasion, migration and drug resistance. Targeting RBFOX2 with compounds, such as T‐1101 tosylate and RNA‐PROTACs, shows promise for treating related diseases. CTD, C‐terminal domain; PRC2, polycomb repressive complex 2; PROTAC, proteolysis‐targeting chimeras; RRM, RNA‐recognition motif. Created with BioRender.com.

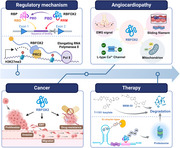

## 1 INTRODUCTION

RNA‐binding proteins (RBPs) play pivotal roles in orchestrating RNA‒protein interactions, governing all facets of RNA metabolism from its inception to its degradation.^1^ These multifaceted proteins harbour diverse RNA‐binding domains (RBDs), enabling them to recognise a spectrum of short RNA elements and auxiliary non‐RBDs.^2^ RBPs exhibit the capability to identify and interact with particular RNA sequences via their respective RBDs. This interaction facilitates a spectrum of crucial cellular processes, encompassing alternative splicing, polyadenylation, nuclear export, intracellular localisation, degradation and regulation of translation.^3^

Within the lineage of evolutionarily conserved RBPs, the RBFOX family emerges as a pivotal regulator, particularly notable for its role in modulating alternative pre‐mRNA splicing. This regulatory function is especially pronounced in the developmental contexts of brain, heart and muscle tissue.^4^ The RBFOX family has been elucidated as a central player in orchestrating the alternative splicing program of numerous genes, underscoring its pivotal role in mediating tissue‐specific regulatory processes.^5^ The gene for one prominent member of this family, RNA‐binding fox‐1 homologue 2 (*RBFOX2*), alternatively known as RNA‐binding motif protein 9 (*RBM9*), is situated on the negative strand of chromosome 22q12.3 (hg38 chr22:35738736‐36028824), encompassing a total gene length of 290 089 bp. Mirroring the behaviour of other RBFOX proteins, RBFOX2 predominantly influences spliced isoform expression through its modulation of alternative splicing events in target gene exons.^4^ Recent investigations have unveiled RBFOX2's pivotal regulatory roles across multiple biological processes, including cell proliferation,^6^ invasion, migration^7^ and excitation‒contraction (E‒C) coupling.^8^ Perturbed RBFOX2 expression has been correlated with the onset and progression of various malignancies^9^ and cardiovascular disorders.^10^ Simultaneously, aberrations in RBFOX2 have been implicated in conferring resistance to anticancer drugs, such as tamoxifen (TAM)^11^ and Rebecsinib.^6^

As a central player in the realm of splicing regulation, a potential biomarker and a therapeutic target for a multitude of disease‐associated genes, RBFOX2 holds substantial promise for research endeavors. While studies pertaining to RBFOX2 are steadily on the rise, there is a conspicuous dearth of comprehensive reviews elucidating its molecular regulatory mechanisms and proposing targeted therapeutic strategies under both physiological and pathological conditions. Consequently, this article aims to systematically synthesise knowledge about RBFOX2, pinpoint the advancements and lacunae in current research, and furnish a directional compass and theoretical framework for ensuing investigations in this field.

## 2 ELUCIDATING THE PROTEIN DOMAIN ARCHITECTURE AND TRANSCRIPT PROFILE OF RBFOX2

**FIGURE 1:**
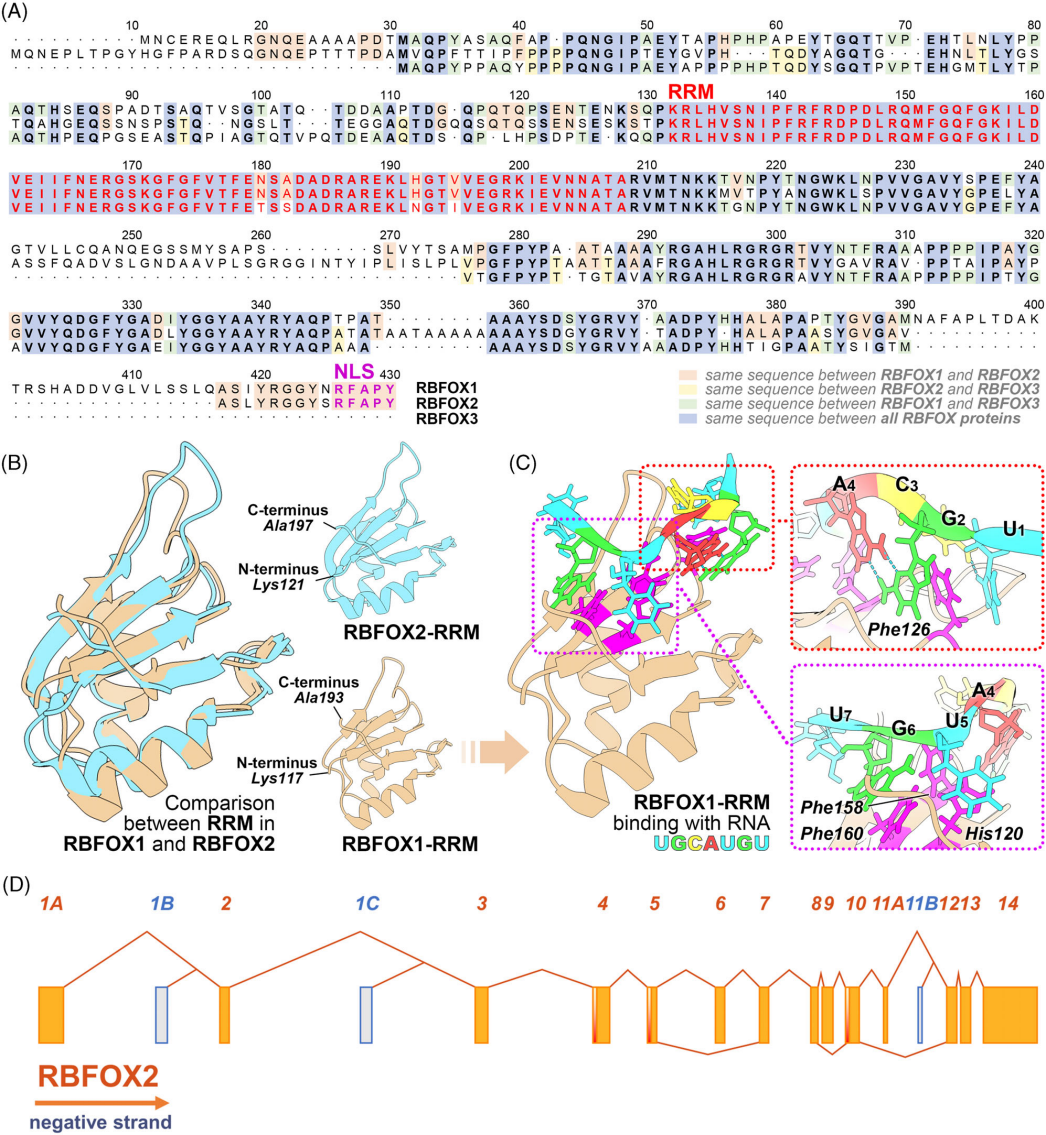
Decoding RNA‐binding fox‐1 homologue 2 (RBFOX2): insights into structure, function and RNA‐binding conformation. (A) Primary structure of RBFOX2: the protein sequence of RBFOX2 is compared with its family proteins RBFOX1 and RBFOX3. Notably, the sequences of the RNA‐recognition motif (RRM) and nuclear localisation signal (NLS) are identical in RBFOX2 and RBFOX1. (B) Comparison of RRM between RBFOX2 and RBFOX1: a detailed comparison of the RRM between RBFOX2 and RBFOX1 highlights their structural similarities. (C) RNA‐binding conformation of RBFOX1 with UGCAUGU motif: the RNA‐binding conformation of RBFOX1 with the UGCAUGU motif is depicted. Within this motif, Phe126 plays a crucial role in binding U1, G2, C3 and A4, while His120, Phe158 and Phe160 are essential for binding U5, G6 and U7. Protein structure data utilised in this analysis is sourced from the Protein Data Bank (2CQ3 and 2ERR) and visualised using ChimeraX.^16^ (D) Various transcripts of human RBFOX2 depict a complex exon organisation, where constitutive exons are delineated by orange boxes, alternative exons by grey‒blue boxes, and regions potentially untranslated by red gradients. Exon numbering serves for precise identification, correlating with genomic coordinates detailed in Table S1. We numbered the RBFOX2 exons with Arabic numerals based on the order of their starting sites on the chromosome. Alternative exons were numbered with letters according to their starting sites on the chromosome. This splicing procedure ensures that each exon has a unique identifier, allowing for a clearer description of the gene structure and alternative splicing forms. It is important to note that this exon numbering may not align with previously reported exon designations (coordinate information of RBFOX2 exons based on GRCh38/hg38).

RBFOX constitutes a class of alternative splicing regulators that have endured through mammalian evolution.^5^ Currently, researchers have identified three proteins within the RBFOX family: RBFOX1, RBFOX2 and RBFOX3.^12^ All members of the RBFOX family share a relatively conserved RNA‐recognition motif (RRM), which exhibits a high‐affinity binding capacity for the hexanucleotide sequence (U)GCAUG (Figure 1).^12^, ^13^ These RRMs are indispensable for the specific recognition of RNA sequences by RBFOX family proteins.^12^ Simultaneously, beyond its recognition of the (U)GCAUG sequence, the RRM of RBFOX has demonstrated an additional capacity to bind to the GCACG sequence. However, it is noteworthy that its binding affinity towards GCACG is comparatively diminished when juxtaposed with (U)GCAUG.^14^ Intriguingly, the functionality of RBFOX proteins extends beyond this motif, as they can be indirectly recruited to RNA through participation in the formation of larger assemblies known as large assembly of splicing regulator (LASR) complexes, and can even exhibit splicing activity in the absence of the (U)GCAUG motif.^15^

Furthermore, within the RBFOX family, it has been observed that both RBFOX1 and RBFOX2 proteins possess a conserved nuclear localisation signal (NLS) situated in the C‐terminal domain (CTD).^13^ This NLS plays a pivotal role in ensuring strong nuclear localisation of RBFOX family proteins under non‐stress conditions.^17^ Under stress conditions, RBFOX family proteins show cytoplasmic localisation mediated by stress granules.^18^ When cells are under stress, numerous proteins and RNAs aggregate in the cytoplasm to form non‐enveloped structures known as stress granules.^19^ RBFOX family proteins strongly bind to stress granules through their RRM, thereby shifting from nuclear to cytoplasmic localisation.^18^ Alterations in the subcellular localisation of these proteins are intimately connected to changes in their capacity to regulate alternative splicing.^20^ It is worth noting that aside from the RRM and NLS regions, the remaining sequences of RBFOX proteins display limited conservation.^13^

In addition to their structural features, RBFOX family coding genes can generate a multitude of distinct transcripts through alternative splicing of RRM coding domains and some exons, which affects their subcellular localisation and regulatory functions in alternative splicing.^21^ *RBFOX2* comprises 14 exons and 13 introns, generating a total of 20 transcripts, 15 of which have protein‐coding potential. Different transcripts can be translated to produce protein isoforms with varying splicing activities. For example, exon 6 encodes 31 of the 73 residues of the RBFOX2 RRM.^22^ Skipping exon 6 eliminates β‐strand 3 and α‐helix 2 from the RBFOX2 structure. β‐Strand 3 contains the key RNP1 motif for RRM folding and has several crucial RNA contact sites.^12^ Thus, exon 6 is key to the structural integrity of RBFOX2 RRM, and its omission will completely alter the protein's conformation and RNA‐binding ability. In‐depth study of the RBFOX2‐ΔRRM isoform revealed that RBFOX2‐ΔRRM not only lost its RNA binding activity but also inhibited the alternative splicing function of other RBFOX2 proteins containing a complete RRM. Therefore, scientists classified these RBFOX2 isoforms that act in a dominant negative (DN) manner as DN subtypes.^22^ Different transcripts also produce protein isoforms with different subcellular localisations. For example, exon 9 contains a 5‐amino acid coding sequence (Ser‐Leu‐Pro‐Leu‐Val) associated with RBFOX2 subcellular localisation. By including this peptide, RBFOX2 can translocate from the nucleus to the cytoplasm.^23^ Deletion of *RBFOX2* exon 10 likely causes a frameshift, resulting in the RBFOX2‐ΔE10 isoform, which contains a PTEVT amino acid sequence at the C‐terminus, causing migration to the cytoplasm.^24^, ^25^ In addition, different transcripts of RBFOX2 are closely related to its indirect binding mode mediated by the LASR complex, which can further affect the splicing activity of RBFOX2. For example, a pair of 43 and 40 nt exons in the CTD coding sequence are specific for encoding muscle (RBFOX2‐43) and non‐muscle isoforms (RBFOX2‐40) of RBFOX2, respectively. The 40 nt exon‐encoded peptide chain contains three tyrosine residues, which are crucial for RBFOX2 to participate in the assembly of LASR.^26^, ^27^ However, muscle isoforms (RBFOX2‐43) lack these tyrosine residues and cannot function through the indirect binding mode, resulting in limited splicing activity.^26^

Distinguished from other RBFOX family proteins, RBFOX2's unique CTD orchestrates the recruitment of the polycomb repressive complex 2 (PRC2) to target genes.^28^ RBFOX2 also acts as a reader recognising specific epigenetic marks on RNA, such as N6‐methyladenosine (m6A)^29^ and 5‐hydroxymethylcytosine (hm5C).^30^ The PRC2‐mediated histone modification (H3K27me3) plays a crucial role in the aberrant silencing of downstream genes^28^ and is a feature observed across diverse cell types in mammals.^31^ When RBFOX2 engages with chromatin‐associated RNAs (caRNAs), it acts as a mediator for the recruitment of the PRC2 complex. Consequently, the PRC2 complex induces histone methylation within the gene promoter region, effectively inhibiting transcription.^28^ In a recent breakthrough, researchers discovered that RBFOX2 recognises m6A modification on caRNAs.^29^ RBFOX2 demonstrates a specific ability to recognise and bind m6A modifications on caRNAs, precisely by virtue of its RRM's recognition of the AGm6AUG sequence.^29^ This event subsequently prompts RBFOX2 to enlist RBM15, a constituent of the methyltransferase complex, to modify promoter‐associated RNAs with m6A. The interaction between RBM15 and the m6A reader YTHDC1 facilitates the recruitment of YTHDC to the site where RBFOX2 binds. Ultimately, YTHDC ushers in the PRC2 complex to the same locus as RBFOX2, thereby instigating H3K27me3 histone modifications at neighboring promoter sites, culminating in transcriptional repression.^28^, ^29^, ^32^ When RBFOX2 acts as a reader of m6A, it plays an important role in acute myeloid leukaemia (AML). Overexpressed RBFOX2 inhibits the expression of *TGFB1* through the RBFOX2/m6A/RBM15/YTHDC1/PRC2 axis, blocks the transforming growth factor‐beta (TGF‐β) signalling pathway, and promotes the progression of AML. Downregulation of RBFOX2 significantly inhibits the survival and proliferation of AML cells and promotes myeloid differentiation. RBFOX2 is essential for the self‐renewal and stemness maintenance of AML cells, making it a potential target for leukaemia treatment.^29^ Additionally, Dou et al. found that RBFOX2 can act as a reader of RNA hm5C, exerting specific recognition and degradation functions on target genes. Specifically, the methyltransferase NSUN5 first acts as a writer to introduce m5C modification on the caRNA of *CTNNB1*, which encodes β‐catenin. NSUN5 then recruits TET2 and RBFOX2 to chromatin. Under the action of TET2, m5C on caRNA is converted to hm5C. Wu et al.’s study first discovered that RBFOX2 can act as a reader of hm5C to inhibit the expression of *CTNNB1*. However, the specific molecular mechanism by which RBFOX2 inhibits the expression of *CTNNB1* has not yet been explored. One possibility is that, similar to its role as a reader of m6A, RBFOX2 regulates the expression of target genes by recruiting the PRC2 complex. This requires further in‐depth research.^30^ This synergistic function of RBFOX2 complements its role in transcriptional repression by recruiting PRC2, indicating that RBFOX2 can act as a multifunctional protein coordinating its interaction with multiple targets.^30^

## 3 UNRAVELING THE VERSATILE FUNCTIONS AND MODES OF ACTION OF RBFOX2 IN ALTERNATIVE SPLICING REGULATION

Alternative splicing is a pivotal determinant of the intricate complexity of multicellular eukaryotic transcriptomes. Its global and gene‐specific regulation governs a wide spectrum of processes, encompassing tissue specificity, cell differentiation and the onset of cancer.^33^ Among the family of RBFOX proteins, RBFOX2, akin to its counterparts, plays a central role primarily within the nucleus as a splicing regulator.^34^

**FIGURE 2:**
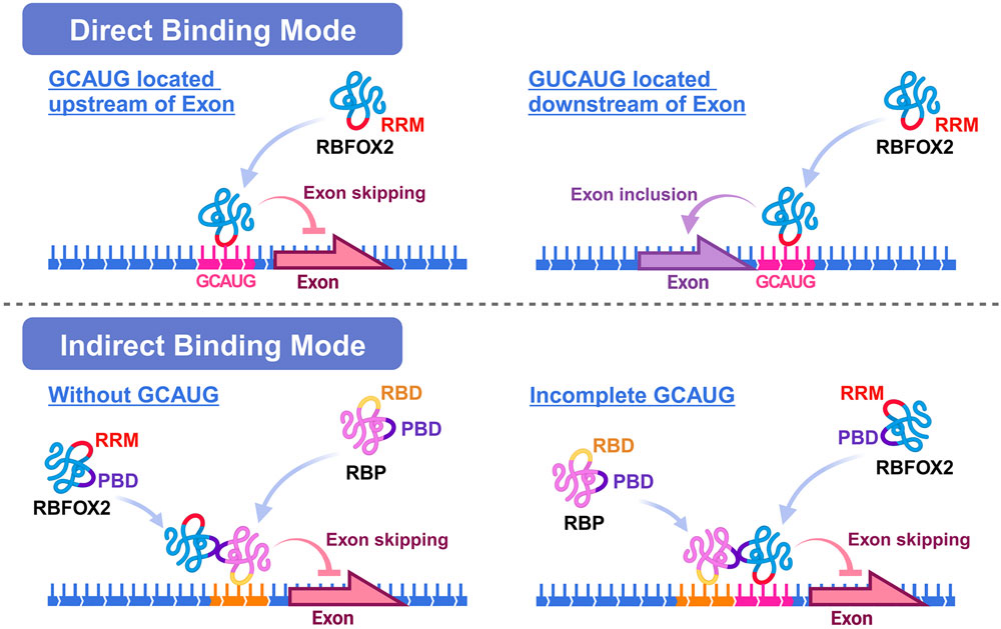
RNA‐binding fox‐1 homologue 2 (RBFOX2) splicing modes: direct and indirect binding mechanisms unraveled. In the direct binding mode, RBFOX2 specifically recognises target RNA sequences containing the (U)GCAUG motif through its inherently conserved RNA‐recognition motif (RRM). When RBFOX2 binds upstream of a target exon splice site, it consistently leads to exon skipping. Conversely, binding downstream of the splice site results in exon inclusion. In the indirect binding mode, RBFOX2 interacts with target RNA indirectly by binding to other RNA‐binding proteins (RBPs). This interaction enables RBFOX2 to cross‐link with the target RNA, allowing it to exert splicing activity regardless of whether the RNA sequence contains the complete (U)GCAUG motif or not. PBD, protein‐binding domain; RBD, RNA‐binding domain; RBP, RNA‐binding protein; RRM, RNA‐recognition motif. Created with BioRender.com.

As detailed in Table 1 and illustrated in Figure 2, recent studies conducted over the past few years have revealed RBFOX2's exceptional capacity to discern unique alternatively spliced RNAs across various tissues.^24^, ^35^ Leveraging its intrinsic RRM and its specific binding to the (U)GCAUG sequence, RBFOX2 can selectively engage target RNA molecules.^12^ Interestingly, not all RBFOX2 splicing targets adhere to the (U)GCAUG motif.^28^ In instances where a complete (U)GCAUG sequence is absent, RBFOX2 employs an indirect approach by recruiting other RBPs to execute splicing functions.^15^, ^36^ Thus, the functions of RBFOX2 are categorised into two distinct modes based on its binding strategies: direct binding modes requiring the (U)GCAUG recognition sequence,^13^ and indirect binding modes that rely on the recruitment by additional proteins.^15^, ^36^

**TABLE 1:** Alternative splicing (AS) function of RNA‐binding fox‐1 homologue 2 (RBFOX2).

Level	Disease	Gene	Exon	AS	Ref.
CVD	Hypertension	*Cacna1c*	9′	Exon skipping	^37^
			33	Exon inclusion	
	Heart failure	*Enah*	13	Exon inclusion	^38^
		*Sorbs2*	4	Exon inclusion	
	Cardiomyopathy	*MICU1*	5′	Exon inclusion	^39^
		*Tpm1*	6a	Exon skipping	^40^
			9b	Exon inclusion	^10^
	CHD	*Atp2b1*	21	Exon inclusion	^41^
		*Mef2a*	9	Exon inclusion	
Cancer	Lymphoma	*ARNT*	5	Exon inclusion	^42^
	NPC	*GOLIM4*	7	Exon inclusion	^32^
	GBM	*PTBP2*	10	Exon inclusion	^43^
	Ewings sarcoma	*ADD3*	14	Exon skipping	^44^
	Endometrial cancer	*TFRC*	4	Exon inclusion	^45^
	Laryngeal cancer	*MENA*	11a	Exon skipping	^46^
	BrC	*NFYA*	3	Exon inclusion	^47^
	BrC/GC	*TEAD1*	6	Exon inclusion	^48^
	HCC	*Numb*	12	Exon inclusion	^49^
		*INSR*	11	Exon inclusion	^50^
	PDAC	*MPRIP*	23	Exon inclusion	^51^
		*MYL6*	6	Exon inclusion	
		*CLSTN1*	10	Exon inclusion	
Others	Hepatitis	*Scarb1*	12	Exon skipping	^52^
	Scleroderma	*LH2*	13A	Exon inclusion	^53^

Abbreviations: BrC, breast cancer; CHD, coronary heart disease; CVD, cardiovascular disease; GBM, glioblastoma multiforme; GC, gastric cancer; HCC, hepatocellular cancer; NPC, nasopharyngeal carcinoma; PDAC, pancreatic ductal adenocarcinoma.

In the direct binding mode, RBFOX2 exhibits a remarkable ability to specifically recognise and bind both primary splicing factor binding sequences (GCAUG) and secondary splicing factor binding sequences (GCCUG or GCUUG) through its conserved RRM, subsequently localising to the target splicing site.^36^, ^51^ RBFOX2 typically targets introns in precursor mRNA (pre‐mRNA),^50^ 3′ untranslated regions (UTRs) in mRNA,^54^ and even pre‐miRNA.^55^ Notably, when RBFOX2 binds downstream binding sites of a target exon, exon inclusion is typically promoted, whereas binding upstream binding sites of a target exon inhibits exon inclusion.^6^ Due to the variable promoter and alternative splicing events that can diversify RBFOX2 gene products, the structure of RBFOX2's RRM is particularly susceptible to disruption. Consequently, RBFOX2 subtypes can be classified into RBFOX2‐wild type (WT) with an intact RRM and RBFOX2‐DN subtypes lacking a complete RRM.^22^ Dysregulation of any RBFOX2 subtype in humans can lead to aberrant splicing in related genes and subsequent disease manifestations.^7^, ^56^ Notably, the isoforms of the RBFOX gene can also be modulated by other RBPs; for instance, overexpression of CELF1 triggers a transition from the RBFOX2‐WT isoform to the RBFOX2‐40 isoform (an RBFOX2‐DN isoform).^26^ Certain subtypes of other RBFOX family members have been observed to influence RBFOX2's RRM, giving rise to RBFOX2‐DN subtypes.^22^, ^57^ In mice, for instance, the Rbfox3 can impede exon 6 inclusion in *Rbfox2* pre‐mRNA, causing an in‐frame deletion of RRM and the consequent emergence of the Rbfox2‐DN isoform.^22^ Certain sno‐lncRNAs, such as SNORD116, exhibit the ability to recruit and form complexes with RBFOX2. This interaction impedes the nuclear migration of RBFOX2, consequently attenuating its role in modulating alternative splicing within the nucleus.^58^

In the indirect binding mode, RBFOX2 forms associations with target RNA indirectly through interactions with other RBPs.^36^ For example, RBFOX2 can be recruited by SRSF1, which binds within or downstream of a target exon, thereby participating in alternative splicing without directly engaging the target RNA.^36^ Similarly, the RBPs recruiting RBFOX2 for indirect RNA binding may also comprise one or more components of a protein complex. Notably, hnRNP M within the LASR complex, a splicing complex, has been identified as an RBFOX2 recruiter, enhancing RBFOX2's binding to target genes.^36^ RBFOX2 can be indirectly recruited to cross‐linking sites by directly binding to hnRNP M as part of the LASR complex,^15^ allowing it to cross‐link with target RNA with or without a complete (U)GCAUG motif and to facilitate splicing activity.^15^ Recent studies have even unveiled RBFOX2's recruitment by the smooth muscle‐specific regulator RBPMS, forming a distinct, cell‐specific splicing regulatory complex distinct from LASR.^59^

Recent studies have shown that in addition to producing different mRNA transcripts through alternative splicing, another outcome of RBFOX2 splicing is the induction of nonsense‐mediated RNA decay (NMD). NMD is an mRNA surveillance pathway that recognises and selectively degrades transcripts containing premature termination codons (PTCs), preventing the production of incomplete or abnormal proteins.^60^ RBFOX2 can introduce PTCs into its downstream mRNA through alternative splicing, causing these mRNAs to be degraded via the NMD pathway. This gene regulation mode, where alternative splicing is coupled with NMD, is also called AS‐NMD.^61^ NMD plays an important role in the expression of RBPs.^62^ As shown in study by Jangi et al., RBPs are particularly enriched in genes regulated by RBFOX2 through AS‐NMD.^61^ These RBPs regulated by RBFOX2 greatly expand its regulatory network. Additionally, since RBFOX2 itself is also an RBP, its mRNA may also be affected by other RBPs in the cell and undergo NMD. This forms a cross‐regulatory competition mechanism between RBFOX2 and other RBPs, wherein they influence each other's expression levels and competitively regulate the expression of downstream genes. This regulatory mechanism increases the complexity of gene expression regulation in cells and may help cells adapt to environmental changes or perform specific functions.^61^, ^63^

## 4 DECIPHERING THE INTRICATE REGULATION OF RBFOX2 SUBCELLULAR LOCALISATION

RBFOX1 and RBFOX2 proteins consistently demonstrate strong nuclear localisation under non‐stressed conditions.^17^, ^21^ This localisation pattern is influenced by alternative splicing events involving the coding sequence of their CTD, giving rise to nuclear localisation isoforms containing NLS and cytoplasmic localisation isoforms devoid of NLS.^4^ However, in contrast to RBFOX1 proteins, the nuclear localisation of RBFOX2 is subject to additional, more stringent regulatory mechanisms beyond its conserved C‐terminal NLS.^25^

RBFOX2 undergoes transcription from at least four distinct promoters, generating an array of isoforms with diverse properties.^64^ Some RBFOX2 isoforms have unique N‐terminal domains (NTD).^25^ When a functional NLS is generated within this unique NTD, it can enable certain subtypes to exhibit nuclear localisation in the absence of a C‐terminal NLS.^25^ For example, in mouse mammary epithelial cells, there are two Rbfox2 isoforms with different NTDs, Rbfox2‐1A and Rbfox2‐1F.^65^ Both isoforms skip or splice exon B40, resulting in frameshifts and the absence of the typical C‐terminal NLS. The NTD of Rbfox2‐1A contains a potential amino acid sequence that functions as a functional NLS, allowing Rbfox2‐1A to show nuclear localisation, unlike Rbfox2‐1F, which shows cytoplasmic localisation.^25^

Furthermore, the subcellular localisation of RBFOX2 can also be influenced by isoforms of other RBFOX family members. For instance, during early chick embryo development, the Rbfox3‐Δ31 isoform, characterised by the exclusion of exon 31aa, is notably abundant in the cytoplasm. This specific isoform, Rbfox3‐Δ31, possesses the ability to modulate the splicing activity of Rbfox2 by altering its subcellular localisation.^34^

Since post‐transcriptional regulation steps usually occur in specific subcellular compartments, such as the nucleus, mitochondria and Golgi apparatus,^66^ abnormal subcellular localisation can theoretically affect the activity of RBFOX2 as a splicing factor in the nucleus, potentially leading to pathological changes.^25^ Under normal nuclear localisation, RBFOX2 regulates RNA splicing by binding to pre‐mRNA, essential for developing and maintaining tissues such as the brain,^35^ muscle^67^ and heart.^68^ The translocation of RBFOX2 to the cytoplasm may regulate its activity as a splicing factor in the nucleus, closely linked to neuronal degeneration,^69^ muscle atrophy^70^ and congenital heart disease.^54^ This translocation is also associated with various malignant phenotypes of tumour cells, such as enhanced cell proliferation, inhibited apoptosis, invasion and migration.^25^, ^51^

## 5 RBFOX2 AND CARDIOVASCULAR DISEASE AND HEALTH

Cardiovascular disease (CVD) stands as a formidable global health challenge, encompassing a spectrum of conditions including cardiomyopathy, coronary heart disease (CHD), hypertension, heart valve disease, arrhythmia, etc.^71^ Recent research endeavours have illuminated RBFOX2's potential utility as a diagnostic and prognostic biomarker for CVDs, with mounting evidence linking its dysregulated expression to the onset of these diseases. RBFOX2, through its intricate molecular mechanisms, contributes significantly to the maintenance of myocardial development and function, playing a pivotal role in the physiological and pathological processes that underpin the cardiovascular system.

### 5.1 RBFOX2 dysregulation in CVDs

The expression profile of RBFOX2 exhibits significant dysregulation across a spectrum of CVDs, as summarised in Table 2. Downregulated RBFOX2 expression characterises conditions, such as dilated cardiomyopathy (DCM)^68^ and CHD.^41^, ^72^ Conversely, diseases marked by elevated RBFOX2 expression include arrhythmia,^26^ myotonic dystrophy type 1 (DM1) cardiomyopathy^26^ and hypertension.^37^ However, the expression of RBFOX2 in diabetic heart disease (DHD)^56^, ^73^ remains subject to controversy.

**TABLE 2:** Dysregulated RNA‐binding fox‐1 homologue 2 (RBFOX2) in cardiovascular diseases.

Disease	Expression of RBFOX2	Case	Control	Ref.
Arrhythmia	Upregulated	3 arrhythmic non‐DM heart samples of DM1 patients	8 autopsied heart samples of normal individuals	^26^
DM1 cardiomyopathy	Upregulated	5 autopsied heart samples of DM1 patients	8 autopsied heart samples of normal individuals	^26^
DCM	Downregulated	Cardiomyocytes from transverse aortic constriction‐treated mice	Cardiomyocytes from sham‐treated mice	^68^
Hypertension	Upregulated	Vascular smooth muscle cells of hypertensive mice	Vascular smooth muscle cells of normal mice	^37^
CHD	Downregulated	Whole heart tissue from CHD in mice (established by streptozotocin)	Whole heart tissue from normal mice (injected daily with citrate buffer)	^41^
	Downregulated	172 surgically discarded cardiovascular tissues	172 surgically discarded cardiovascular tissues	^72^
DHD	Downregulated	Cardiomyocytes from diabetic mice (fed with high‐fat diet)	Cardiomyocytes from normal mice (fed with standard diet)	^56^
	Upregulated	Cardiomyocytes from type 2 diabetic human left ventricles	Cardiomyocytes from non‐diabetic human left ventricles	^73^

Abbreviations: CHD, coronary heart disease; DCM, dilated cardiomyopathy; DHD, diabetic heart disease; DM1, myotonic dystrophy type 1.

In a CHD model established using streptozotocin, Rbfox2 expression is significantly reduced in whole heart tissues.^41^ Similar observations are made in cardiovascular tissues from CHD patients, where RBFOX2 expression is markedly decreased compared to normal samples.^72^ Wei et al. deleted the *Rbfox2* gene by crossing 129S6/SVEvTac mice, with LoxP sites flanking *Rbfox2* exons 6 and 7, with Mlc2v‐Cre transgenic mice, using the Cre‐LoxP recombinase system. Compared to normal mice, the heart sections of *Rbfox2* conditional deletion mice exhibited significant ventricular enlargement and thinning of the ventricular wall, ultimately developing into typical DCM. Notably, their results confirmed that *Rbfox2* deletion leads to DCM, which subsequently causes heart failure, indicating that downregulation of Rbfox2 is not merely a functional consequence of heart failure.^68^

Notably, the upregulation of RBFOX2 is often associated with a specific isoform, the RBFOX2‐DN isoform. In DHD, RBFOX2 expression is elevated in left ventricular cardiomyocytes of human patients with type 2 diabetes mellitus, and this upregulation coincides with increased expression of RBFOX2‐DN isoforms.^73^ Similarly, Rbfox2 expression is increased in rat vascular smooth muscle cells (VSMCs) and upregulated in hypertensive arteries, accompanied by enhanced expression of DN subtypes.^37^ In heart samples from patients with DM1 cardiomyopathy or DM1 non‐diabetic arrhythmia, RBFOX2 upregulation is coupled with the selective upregulation of the RBFOX2‐40 isoform (a type of RBFOX2‐DN).^26^

However, paradoxically, a recent study reveals that Rbfox2 expression is downregulated in the heart tissue of diabetic rats induced by high‐fat feeding compared to the control group.^56^ Despite this downregulation, the levels of Rbfox2‐DN exhibit a clear upward trend, aligning with previous findings.^73^ This suggests that upregulation of RBFOX2‐DN isoforms is a consistent phenomenon in diabetic hearts. Nonetheless, the overall up‐ or downregulation of RBFOX2 levels may be governed by changes in other isoforms, influenced by distinct disease states.

Furthermore, the subcellular localisation of RBFOX2 varies within different cell types. In isolated rat VSMCs and rat mesenteric artery smooth muscle cells, Rbfox2 predominantly localises to the nucleus.^37^ In contrast, in hypoplastic left heart syndrome (HLHS), RBFOX2 is primarily cytoplasmic in the hearts of HLHS patients.^54^ Thus, the ectopic expression of RBFOX2 emerges as a hallmark feature of cardiovascular cytopathies.

### 5.2 RBFOX2 affects cardiovascular health and disease

**FIGURE 3:**
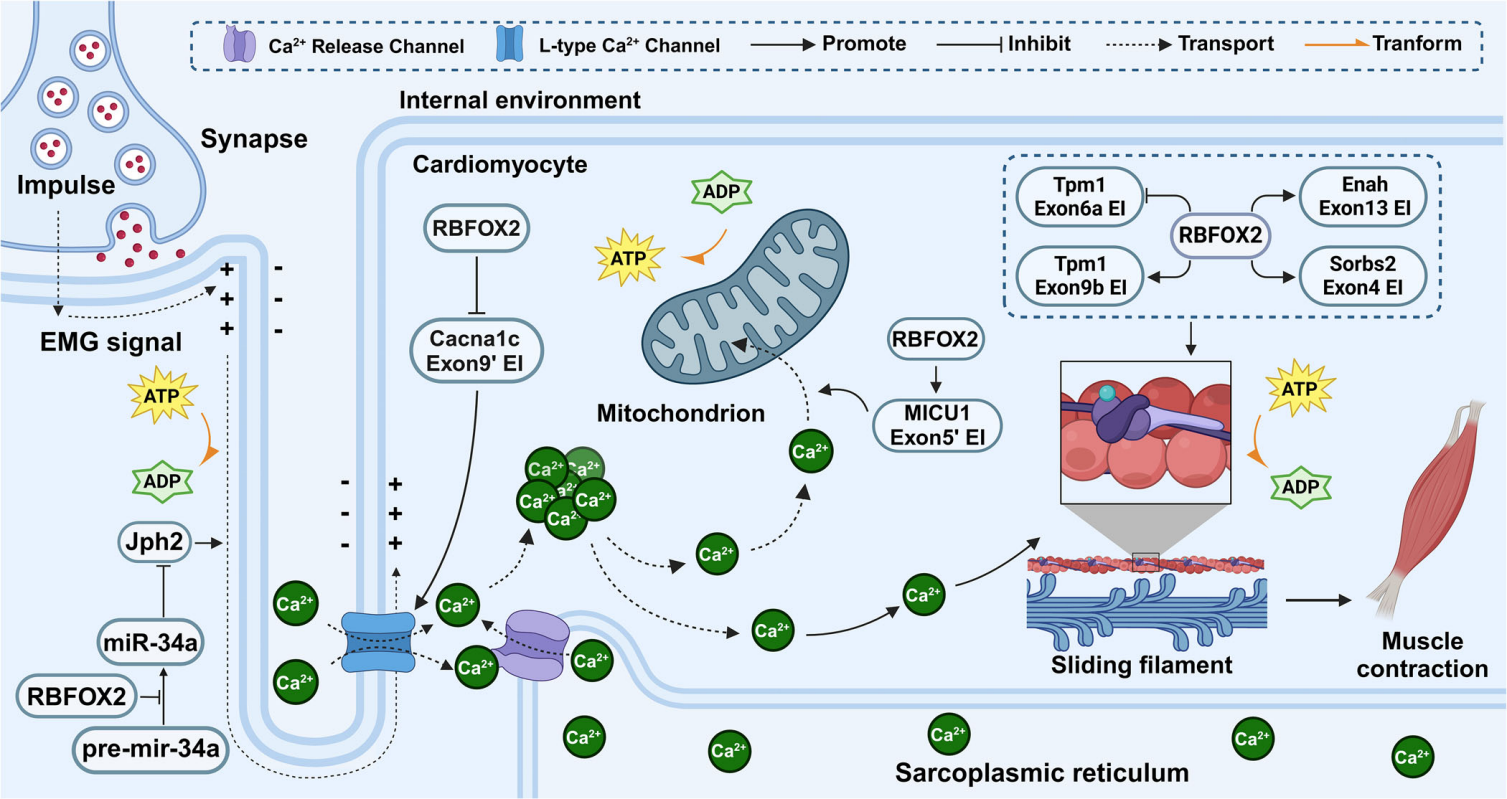
RNA‐binding fox‐1 homologue 2 (RBFOX2): orchestrating cardiomyocyte function. RBFOX2 plays a pivotal role in regulating physiological mechanisms closely associated with cardiomyocyte function, such as excitation‒contraction coupling (E‒C coupling), calcium ion channels and mitochondrial metabolism, by influencing the alternative splicing of downstream genes. Dysregulation of RBFOX2 has been implicated in the onset of various cardiovascular diseases, including arrhythmias and cardiomyopathy. Specifically, RBFOX2 can promote the expression of *Jph2* by inhibiting the maturation of *miR‐34a*, thereby enhancing the conduction of electrical signals in cardiomyocytes. RBFOX2 also inhibits the inclusion of *Cacna1c* exon 9′, thereby stabilising the number of L‐type calcium channels in cardiomyocyte membranes and promoting calcium transients after depolarisation. Additionally, RBFOX2 promotes the inclusion of *MICU1* exon 5′, enhancing mitochondrial Ca^2+^ uptake and thereby boosting energy metabolism. RBFOX2 also facilitates the inclusion of *Enah* exon 13, *Sorbs2* exon 4 and *Tpm1* exon 9b, while inhibiting the inclusion of *Tpm1* exon 6a, thus promoting muscle fibre contraction. EI, exon inclusion; EMG, electromyographic; ES, exon skipping. Created with BioRender.com.

Cardiac dysfunction constitutes a significant contributor to CVDs, encompassing a spectrum of issues ranging from L‐type calcium channel Ca_V_1.2 function to defects in cardiac E‒C coupling and mitochondrial integrity (Figure 3).^56^, ^74^ An increasing body of research underscores the pivotal role of RBFOX2 as a key regulatory factor in myocardial E‒C coupling, Cav1.2 channel function, and the maintenance of mitochondrial morphology. RBFOX2 emerges as a critical player in preserving normal myocardial development and overall cardiovascular health.

Cardiac E‒C coupling orchestrates the transition from electrical excitation to muscle fibre contraction, underpinning proper cardiac function.^8^ While *Jph2* variants have been linked to diverse cardiac disorders, the mechanisms underlying these associations have remained elusive.^75^, ^76^ Recent findings in neonatal rat cardiomyocytes shed light on this conundrum, demonstrating that reduced Rbfox2 levels trigger the maturation of *miR‐34a*. *miR‐34a*, in turn, targets and inhibits the expression of *Jph2*, resulting in E‒C coupling defects that culminate in impaired cardiac contraction and, ultimately, heart failure.^8^, ^38^

The Cav1.2 channel, encoded by the *CACNA1C* gene, represents a critical substrate for cardiac electrophysiological activity.^77^ It participates in E‒C coupling, modulates action potential duration, regulates cardiac cell gene expression and plays a central role in cardiac function.^78^ Aberrant Cav1.2 channel activity can induce premature depolarisation of cardiomyocytes, leading to severe arrhythmias and sudden cardiac death.^79^ In hypertensive rat VSMCs, elevated Rbfox2 inhibits the inclusion of *Cacna1c* exon 9′ and promotes the inclusion of exon 33. Notably, *Cacna1c* exon 9′ and exon 33 exert differential regulation over various biochemical and physiological functions of *Cacna1c*.^80^, ^81^, ^82^ Furthermore, exon 9′ has been shown to play a pivotal role in the contraction of cerebral arteries.^83^ Consequently, dysregulation of Rbfox2's influence on Cav1.2 channel function emerges as a key contributor to hypertension pathogenesis.^37^ *Cacna1c* is the gene encoding Cav1.2, a critical subtype of the L‐type calcium channel in cardiomyocytes. In diabetic rats, when RBFOX2 is downregulated by advanced glycation end‐products, it promotes the inclusion of *Cacna1c* exon 9′ and the expression of Cav1.2. Upon sarcolemma depolarisation, Ca^2+^ enters the cell through membrane‐bound Cav1.2, inducing calcium‐induced calcium release from the sarcoplasmic reticulum into the cytoplasm. Downregulation of RBFOX2 leads to excessive Cav1.2 on the membrane, resulting in excessive Ca^2+^ in the sarcoplasm, continuous contraction of muscle fibres, and subsequent myocardial hypertrophy in diabetic patients.^56^

Mitochondria are pivotal in sustaining cardiac energy metabolism, with preserving their intact morphology forming the bedrock of their functionality.^84^ Mitochondrial abnormalities lead to energy deficits in cardiac tissue, precipitating bioenergetic diseases such as heart failure.^85^ Research has elucidated that in H9c2 cells, the gene *Slc25a4* possesses two distinct polyadenylation sites, resulting in the generation of mRNA molecules with differing lengths of 3′ UTR. Notably, the mRNA variant featuring an extended 3′ UTR exhibits diminished stability and translation efficiency, thereby impeding *Slc25a4* expression. The protein Rbfox2 intervenes in this process by counteracting the influence of the distal polyadenylation site. Through its binding affinity to the UGCAUG motif downstream of the distal polyadenylation site on *Slc25a4*, Rbfox2 prompts the preferential production of mRNA with a shorter 3′ UTR. Diminishment of Rbfox2 levels results in an elevation of *Slc25a4* mRNA harbouring the longer 3′ UTR, consequently dampening Slc25a4 expression. This dysregulation culminates in mitochondrial dysfunction within H9c2 cells, a phenomenon closely linked to cardiomyopathy pathogenesis.^10^, ^86^ Moreover, during cardiac differentiation, RBFOX2 selectively promotes the inclusion of *MICU1* exon 5′, endowing specificity to mitochondrial Ca^2+^ uptake, a critical component for ATP production during muscle contraction.^39^, ^87^

In addition, multiple splicing events coordinated by Rbfox2 contribute to myoblast fusion. For example, during myocyte differentiation, Rbfox2 promotes the production of two muscle‐specific isoforms (Mef2d and Rock2) that synergistically control the terminal differentiation of myoblasts into multinucleated myotubes.^88^, ^89^

In heart failure, reduced mouse Rbfox2 expression inhibits the inclusion of exon 13 in *Enah* and exon 4 in *Sorbs2*, while during heart development,^38^ mouse Rbfox2 governs the balance by repressing *Tpm1* isoforms containing exon 6a^40^ and promoting *Tpm1* isoforms with exon 9b.^10^ Dysregulated splicing patterns of key genes involved in the cGMP‒PKG‒Ca^2+^ pathway, such as *Atp2b1* exon 21 and *Mef2a* exon 9, heighten the risk of cardiomyopathy and heart failure in patients with CHD and diabetes.^41^ In diabetic mouse foetal hearts, the inhibition of the PKC‐mediated activation of Rbfox2 disrupts early developmental homeostasis.^90^ Additionally, RBFOX2 influences the alternative splicing of genes like *MBNL1*, *MCAM*, *VCL* and *ACTN1*, with downstream effects on the differentiation and function of cardiac smooth muscle cells.^91^ It is worth noting that the Gazzara et al. confirmed the splicing antagonism between CELF2 and the RBFOX family through knockdown and overexpression experiments in human cells, showing that CELF2 inhibits *RBFOX2* mRNA and protein levels.^92^ Both CELF2 and RBFOX proteins are involved in the development and maintenance of neurons, muscle and cardiac tissues, and their antagonism contributes to splicing regulation in normal cardiac development and disease.^57^, ^93^ However, the crosstalk between these proteins has not been directly studied. A deeper understanding of CELF2 and RBFOX2's functional antagonism may explain differences in expression and splicing between muscle and brain.

Given its pivotal role in cardiovascular health, the dysregulation of RBFOX2 can lead to a range of CVDs, including cardiomyopathy^10^ and heart failure.^38^ Antagomir, a synthetic miRNA inhibitor capable of binding to specific miRNAs, has shown promise in ameliorating heart failure characterised by low Rbfox2 expression. Multiple upregulated cardiac key miRNAs (*let‐7f*, *miR‐16*, *miR‐200b*) in heart failure negatively regulate *Rbfox2*. However, antagomir cocktail therapy has demonstrated the potential to restore Rbfox2 levels in a mouse cardiomyocyte‐induced heart failure model.^38^ Consequently, antagomir presents a potential therapeutic avenue for treating heart failure with a low expression of RBFOX2 phenotype.^38^

## 6 DYSREGULATED RBFOX2 IN CANCER PROGRESSION

Cancer stands as the foremost contributor to global mortality.^94^ Within the realm of cancer, RBFOX2 emerges as a potential biomarker, wielded for both diagnostic and prognostic purposes, while its aberrant expression forms an intricate association with the extent of tumour malignancy. Furthermore, RBFOX2 exhibits the capability to foster the phenotypic traits of cancer cells and steer the inexorable march of tumour progression. Notably, RBFOX2 plays a critical role in bolstering the viability of cancer cells even following treatment, thereby constituting a substantial contributing factor to the development of tumour resistance against anticancer therapies.

### 6.1 RBFOX2 dysregulation and subcellular localisation as diagnostic and prognostic indicators in cancer

In the context of cancer, RBFOX2 undergoes dysregulation at various levels within cells and tissues. Aberrant RBFOX2 expression serves as a valuable biomarker for assessing the malignancy of cancer. Additionally, abnormal subcellular localisation of RBFOX2 holds promise for evaluating the degree of malignancy. As presented in Table 3, cancer types exhibiting elevated RBFOX2 expression encompass lymphoma,^42^ uveal melanoma (UM),^95^ laryngeal cancer,^46^ nasopharyngeal carcinoma (NPC),^32^ gastric cancer (GC),^96^ colorectal cancer (CRC)^97^ and breast cancer (BrC).^7^, ^98^ Conversely, cancers with reduced RBFOX2 expression include glioblastoma multiforme (GBM)^43^ and pancreatic ductal adenocarcinoma (PDAC).^51^

**TABLE 3:** Dysregulated RNA‐binding fox‐1 homologue 2 (RBFOX2) in cancers.

System	Disease	Expression of RBFOX2	Level	Case	Control	Ref.
Blood system	Lymphoma	Upregulated	Cell	Karpas299 and Jurkat	Primary human T cells	^42^
	AML	Downregulated	Cell	Paediatric AML‐derived HSCs/HPCs	Non‐leukaemic HSCs/HPCs	^6^
		Upregulated	Tissue	427 bone marrow samples from GEO database (GSE13159)	73 healthy bone marrow samples from GEO database (GSE13159)	^29^
			Cell	MM6, MOML13, NOMO1, NB4 and K562	CD34^+^ HSCs/HPCs from normal human umbilical cord blood	
Nervous system	UM	Upregulated	Tissue	56 UM tissues from the TCGA database and 26 UM tissues from the Rotterdam Ocular Melanoma Study Group	56 UM tissues from the TCGA database and 26 UM tissues from the Rotterdam Ocular Melanoma Study Group	^95^
	GBM	Downregulated	Tissue	66 anaplastic oligodendroglioma tissues, 145 anaplastic astrocytoma tissues, and 216 GBM tissues from the Brain Tumour Biorepository of the University of Alabama	21 normal brain tissues from the Brain Tumour Biorepository of the University of Alabama	^43^
Respiratory system	Laryngeal cancer	Upregulated	Cell	Tu177, Tu212, M4E and Hep2	NP69	^46^
	NPC	Upregulated	Tissue	Tumour tissues from 20 NPC individuals in Sun Yat‐Sen University Cancer Center	Rhinitis tissues from 19 rhinitis individuals in Sun Yat‐Sen University Cancer Center	^32^
Digestive system	GC	Upregulated	Cell	SGC7901, MGC803, BGC823 and AGS	GES1	^96^
	CRC	Upregulated	Tissue	50 CRC tumour tissue samples	50 normal tissue samples	^97^
		Upregulated	Tissue	42 CRC tissue samples including from TCGA database	54 normal tissue samples from TCGA database	^99^
	PDAC	Downregulated	Tissue	PDX model established by injecting metastatic tumour tissue into NOD‐SCID mice	PDX model established by injecting primary tumour tissue into NOD‐SCID mice	^51^
		Upregulated	Tissue	45 PDAC tissue samples from GEO database (GSE28735)	45 normal tissue samples from GEO database (GSE28735)	^100^
Reproductive system	BrC	Upregulated	Cell	MCF7 and HCC1806 in oxygen deficit condition	MCF7 and HCC1806	^101^
		Upregulated	Tissue	157 TNBC tissues from TCGA database	774 other types of BrC tissues from TCGA database	^7^

Abbreviations: AML, acute myeloid leukaemia; BrC, breast cancer; CRC, colorectal cancer; GBM, glioblastoma multiforme; GC, gastric cancer; HPC, haematopoietic progenitor cell; HSC, haematopoietic stem cell; NOD‐SCID, non‐obese diabetic/severe combined immunodeficiency; NPC, nasopharyngeal carcinoma; PDAC, pancreatic ductal adenocarcinoma; PDX, patient‐derived xenograft; TNBC, triple‐negative breast cancer; UM, uveal melanoma.

In lymphoma, RBFOX2 expression significantly surpasses that in primary human T cells, as evidenced by its higher levels in human lymphoma cells (Karpas299 and Jurkat).^42^ Within the UM cohort, analysis of data from TCGA and ROMScohort reveals heightened RBFOX2 expression in patients at higher risk.^95^ Laryngeal carcinoma cells (Tu177, M4E, Hep2 and Tu212) show elevated RBFOX2 expression relative to normal nasopharyngeal epithelial cells (NP69) in laryngeal cancer.^46^ Furthermore, tumour tissues from NPC patients exhibit significantly higher RBFOX2 levels than mucosal tissues from rhinitis patients.^32^ GC cell lines (MCF‐10ASGC7901, MGC803, BGC823 and AGS) manifest increased RBFOX2 expression compared to human normal gastric mucosal epithelial cells (GES1).^96^ In CRC, RBFOX2 registers upregulation in cancer tissues compared to normal counterparts.^97^ Similarly, in BrC, hypoxia‐treated cell lines (MCF7 and HCC1806) display higher RBFOX2 expression than their normoxia‐treated counterparts. In more malignant BrC patients, specifically those with triple‐negative breast cancer (TNBC), RBFOX2 expression significantly exceeds that in other BrC patients, as indicated by TCGA data.^7^, ^101^ In contrast, within the context of PDAC, patient‐derived xenograft (PDX) models from metastatic PDAC display reduced RBFOX2 expression relative to PDX models derived from primary PDAC tumour tissue.^51^ Moreover, in glioma, RBFOX2 expression is lower in lesion brain tissue of glioma patients compared to normal brain tissue, and within glioma patients, those with higher malignancy exhibit lower RBFOX2 expression in lesion brain tissue than those with less malignancy.^43^

The abnormal expression pattern of RBFOX2 in AML remains uncertain. A study utilising the GEO database (GSE13159) revealed that AML patients across various subtypes, including normal karyotype, mixed lineage leukaemia and t(15:17), exhibited significantly elevated RBFOX2 expression in bone marrow samples compared to healthy counterparts. Additionally, Western blot analysis demonstrated higher RBFOX2 expression in five different types of AML cell lines (MM6, MOML13, NOMO1, NB4 and K562) compared to normal human myeloid cell line CD34^+^ HSCs/HPCs.^29^ However, contrasting findings emerged from studies employing fluorescence‐activated cell sorting, indicating that paediatric AML‐derived haematopoietic stem cells (HSCs) and haematopoietic progenitor cells (HPCs) expressed lower levels of RBFOX2 than their cord blood‐derived counterparts.^6^ The observed differential expression patterns of RBFOX2 in paediatric AML and adult AML may stem from variances in the splicing events occurring within these distinct contexts.^6^ These conflicting results underscore the complexity of RBFOX2 expression regulation in AML and highlight the need for further research to elucidate its role in this context.

It is important to note that RBFOX2 predominantly localises within the nucleus, although its cytoplasmic localisation appears to be cancer cell‐specific. For instance, in CRC, normal colon tissues exhibit nuclear RBFOX2 localisation, whereas strong cytoplasmic RBFOX2 staining is observed in various regions of CRC tissues. Quantitative assessment of nucleoplasmic distribution of RBFOX2 in cells further demonstrates significant differences between normal colon tissue and colon cancer tissue.^97^

Furthermore, as delineated in Table 4, RBFOX2 emerges as an independent adverse prognostic factor in various cancer types, including AML, NPC, UM and GC. AML patients exhibiting high RBFOX2 expression demonstrate poorer overall survival.^29^ High RBFOX2 expression in NPC is associated with diminished overall survival and disease‐free survival.^32^ In UM, elevated RBFOX2 expression correlates with worsened overall survival.^95^ In GC, heightened RBFOX2 expression links to advanced clinical and TNM stages, as well as inferior overall survival.^96^, ^102^ Conversely, in other cancers such as PDAC, hepatocellular cancer (HCC) and GBM, the prognostic implications of RBFOX2 dysregulation contrast with the aforementioned findings. PDAC patients displaying low RBFOX2 expression experience inferior overall survival.^51^ Similarly, HCC patients exhibiting low RBFOX2 expression have a poorer overall survival,^49^ and GBM patients with reduced RBFOX2 expression encounter diminished overall survival.^43^

**TABLE 4:** The role of RNA‐binding fox‐1 homologue 2 (RBFOX2) in tumour progression.

System	Disease	Effects in vitro	Related cell lines	Effects in vivo	Cancer models	Prognosis	Case	Ref.
Blood system	AML	Proliferation↓	Paediatric AML‐derived HSCs and HPCs	–	–	–	–	^6^
		Differentiation↓	NB4	Differentiation↓	CDX model established by injecting MOLM13 cells into NRG‐SGM3 mice	Worse OS	106 AML patients from TCGA database	^29^
Motor system	Ewings sarcoma	EMT↑	ASP14	–	–	–	–	^44^
Nervous system	GBM	Proliferation↓	U87MG	–	–	Better OS	397 glioma patients from TCGA database	^43^
		EMT↑	GSC1023 and GSC0910 (glioblastoma stem cell lines derived from GBM patients)	–	–	–	–	^103^
Respiratory system	Laryngeal cancer	Proliferation↑, EMT↑ and invasion↑	Tu177 and Hep2	–	–	–	–	^46^
	NPC	Proliferation↑ and invasion↑	S26 and 5‐8F	Growth↑	CDX model established by injecting S26 cells into BALB/c nude mice	Worse OS and DFS	85 NPC patients from Sun Yat‐Sen University Cancer Center	^32^
Digestive system	PDAC	Invasion↓ and migration↓	BxPC3	Metastasis↓	PDX model established by injecting metastatic tumour tissue into NOD‐SCID mice	Better OS	136 primary tumour samples from Sheba Medical Center	^51^
		Invasion↑ and migration↑	MiaPaCa2, 4039, and Panc1	–	–	–	–	^100^
	CRC	Invasion↑ and migration↑	HCT116	–	–	–	–	^99^
	HCC	–	–	–	–	Better OS	241 HCC patients from Liver Cancer Institute, Zhongshan Hospital, and Beijing 302 Hospital	^49^
	GC	Proliferation↑	SGC7901 and MGC803	–	–	Worse OS	92 GC patients from the First Affiliated Hospital of Guangzhou University	^96^
		Proliferation↑, invasion↑ and migration↑	AGS and MKN28	Growth↑ and metastasis↑	CDX model established by injecting MKN45 cells into BALB/c nude mice	Worse OS	50 GC patients from the First Affiliated Hospital of Sun Yat‐sen University	^102^
Reproductive system	Endometrial cancer	Ferroptosis↑	KLE and Ishikawa	–	–	–	–	^45^
	Ovarian cancer	Apoptosis↓	HEY and OVARY1847	–	–	–	–	^104^
	BrC	EMT↑ and invasion↑	MCF‐7 and HCC‐1806	–	–	–	–	^101^
		Invasion↑ and migration↑	MCF‐7 and MDA‐MB‐231	–	–	–	–	^7^
		Invasion↑	MCF‐7 and MDA‐MB‐231	–	–	–	–	^105^

Abbreviations: AML, acute myeloid leukaemia; BrC, breast cancer; CDX, cell‐derived xenograft; CRC, colorectal cancer; DFS, disease‐free survival; EMT, epithelial‒mesenchymal transition; GBM, glioblastoma multiforme; GC, gastric cancer; HCC, hepatocellular cancer; NOD‐SCID, non‐obese diabetic/severe combined immunodeficiency; NPC, nasopharyngeal carcinoma; OS, overall survival; PDAC, pancreatic ductal adenocarcinoma.

### 6.2 RBFOX2 drives tumour progression

**FIGURE 4:**
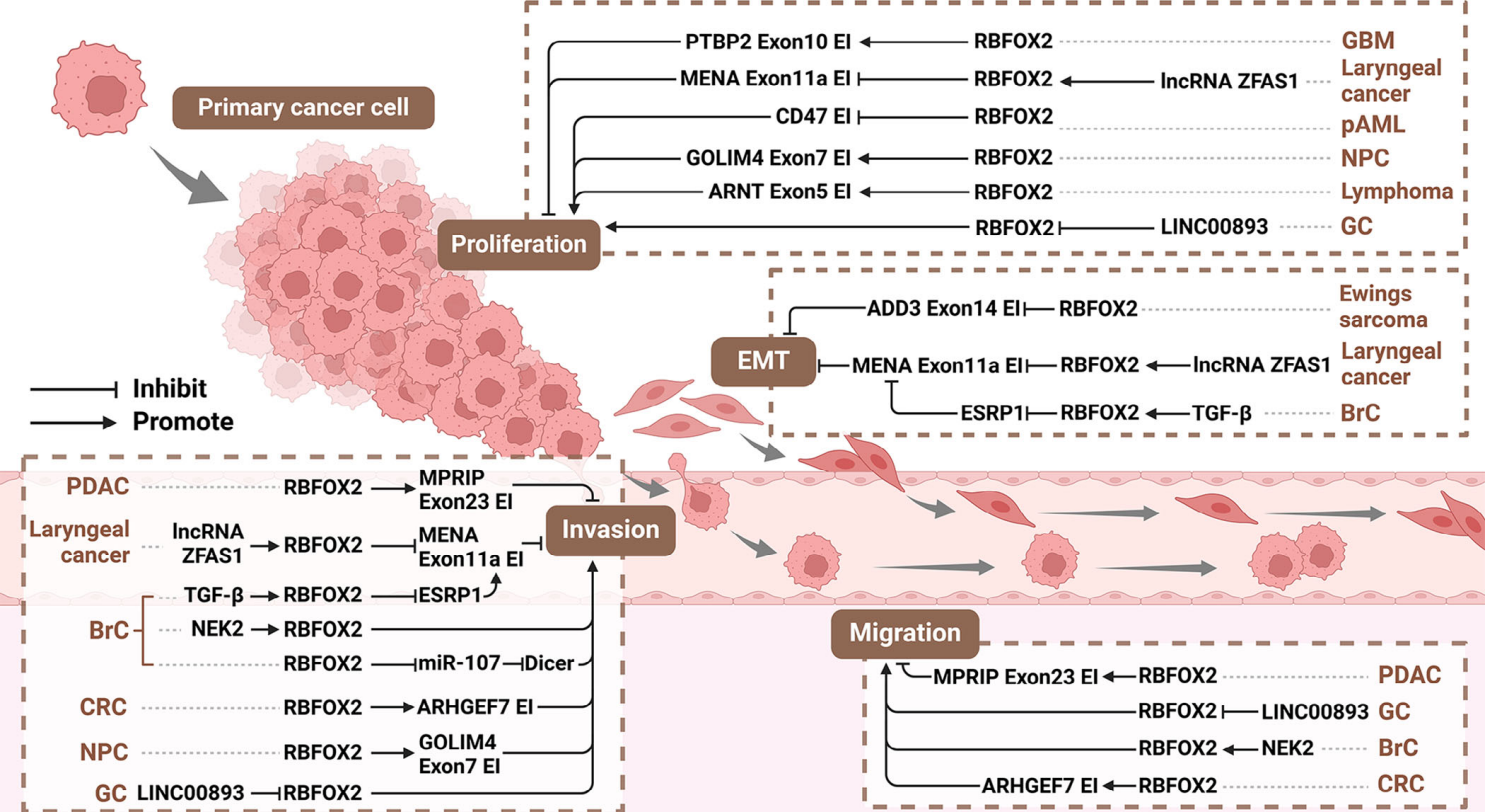
RNA‐binding fox‐1 homologue 2 (RBFOX2): master regulator of cancer cell behaviour. RBFOX2 exerts control over the biological behaviours of diverse cancer cells, including proliferation, epithelial‒mesenchymal transition (EMT), invasion and migration. It achieves this by directly or indirectly modulating the alternative splicing of genes associated with cancer. Imbalances in RBFOX2 expression are implicated in the onset and progression of cancers across various systems, including digestive, respiratory and haematological systems. BrC, breast cancer; CRC, colorectal cancer; EI, exon inclusion; ES, exon skipping; GBM, glioblastoma multiforme; GC, gastric cancer; NPC, nasopharyngeal carcinoma; pAML, paediatric acute myeloid leukaemia; PDAC, pancreatic ductal adenocarcinoma. Created with BioRender.com.

The uncontrolled proliferation and metastasis of tumour cells within the human body represent the primary challenges in the quest to conquer cancer.^106^ In recent years, mounting evidence underscores the pivotal role of RBFOX2 in tumour progression. As depicted in Table 4 and Figure 4, RBFOX2 exhibits a nuanced influence on cancer, sometimes promoting tumour proliferation and metastasis, while in other instances, inhibiting these processes.

BrC stands out as a highly heterogeneous tumour arising from mammary duct epithelial cells, distinguished by its robust invasive and metastatic potential.^107^, ^108^ Within BrC cell lines (MCF‐7, HCC‐1806 and MDA‐MB‐231), RBFOX2 significantly bolsters the invasion and migration of cancer cells.^7^, ^98^, ^105^ In hypoxic conditions, TGF‐β signalling upregulates RBFOX2 in BrC cells (MCF‐7 and HCC‐1806), thereby inhibiting *ESRP1* at the transcriptional level, consequently fostering the pro‐metastatic MENAΔ11a subtype, enhancing epithelial‒mesenchymal transition (EMT) in BrC cells, and augmenting invasiveness.^101^ A lower ESRP1/RBFOX2 ratio has been correlated with increased metastatic risk in BrC patients.^82^ Additionally, RBFOX2 modulates Dicer expression via direct interaction with *pre‐miR‐107*, inhibiting its maturation and subsequently increasing the invasiveness of BrC cell lines.^105^ Furthermore, in the more aggressive TNBC, the oncogenic kinase NEK2 stimulates the expression and activity of RBFOX2, thereby promoting the migratory and invasive phenotypes of TNBC cell lines (MDA‐MB‐231).^7^

In various other cancer types, RBFOX2 has displayed similar tumour‐promoting functions. For instance, in AML mouse bone marrow cells (MLL‐AF9) and PDX models, Rbfox2 inhibits bone marrow stem cell differentiation.^29^ In in vitro cultures of GC cell lines (SGC7901 and MGC803), RBFOX2 overexpression has been shown to enhance the malignant characteristics of GC cells by activating the PI3K/AKT and MAPK signalling pathways.^96^ Another study conducted with GC cell lines (AGS and MKN28) revealed that *LINC00893* directly binds to RBFOX2, inducing its ubiquitination and degradation. This interaction led to the inhibition of proliferation, migration and invasion of GC cells.^102^ Additionally, in a cell‐derived xenograft (CDX) model constructed from MKN45, a GC cell line derived from the gastric lymph nodes of female signet ring cell carcinoma patients, RBFOX2 was found to promote tumour growth and metastasis.^102^ These findings underscore the multifaceted role of RBFOX2 in GC progression, shedding light on potential therapeutic avenues. In CRC cells (HCT116), RBFOX2 directly binds to *ARHGEF7* pre‐mRNA, promoting the inclusion of cancer‐associated micro‐exons and promoting tumour invasion and migration.^99^ In NPC cells (S26 and 5‐8F), RBFOX2 boosts the expression of *GOLIM4‐L* by promoting the inclusion of *GOLIM4* exon 7, subsequently enhancing the proliferation and migration of NPC cells in vitro and facilitating CDX model growth in vivo.^32^ In laryngeal cancer cells (Tu177 and Hep2), the upregulated lncRNA *ZFAS1* binds to and upregulates RBFOX2 expression. Elevated RBFOX2 promotes the skipping of *MENA* exon 11a, ultimately stimulating proliferation, EMT, and invasion of laryngeal cancer cells.^46^ In ovarian cancer, the lncRNA *MALAT1* has been identified to enhance the expression of RBFOX2, subsequently suppressing apoptosis in ovarian cancer cells (HEY and OVARY1847).^104^ Moreover, in the Ewing's sarcoma cell line (A673), RBFOX2 has been found to bind to *ADD3* (a phenotypic driver of Ewing's sarcoma) pre‐mRNA. This interaction inhibits the inclusion of *ADD3* exon 14, promoting the mesenchymal phenotype of Ewing's sarcoma cells.^44^ These insights illuminate the intricate regulatory roles of RBFOX2 in different cancer contexts, providing valuable clues for targeted therapeutic interventions.

In contrast to these findings, some studies reveal that RBFOX2 can also exert inhibitory effects on tumour proliferation and invasion. For example, CD47, a receptor widely expressed in cancer cells facilitating evasion from immune system phagocytosis, experiences downregulation of splice isoforms through RBFOX2 in paediatric AML‐derived HSCs/HPCs, subsequently impeding tumour proliferation.^6^ In PDAC cells (BxPC3), RBFOX2 has been identified as a promoter of *MPRIP* exon 23 inclusion. This inclusion enhances the capacity of *MPRIP* to inhibit RHOA through its RHO GTPase activating protein function, thereby suppressing tumour cell invasion, migration and in vivo metastasis as demonstrated in a PDX model.^51^ Recent studies have found that in PDAC, downregulation of RBFOX2 can promote skipping of *ABI1* exon 9, causing ABI1 to redistribute from the cytoplasm to the cell periphery. ABI1 at the periphery participates in assembling the WAVE signalling complex, promoting actin polymerisation and cytoskeleton remodeling by activating the Arp2/3 complex, thereby enhancing cell migration and invasion, and causing chemotherapy resistance.^100^, ^109^ These findings underscore the significant impact of RBFOX2‐mediated splicing regulation on the invasive behaviour of PDAC cells, providing potential avenues for therapeutic intervention.

Moreover, in GBM cells (U87MG), RBFOX2 counters *SON* by promoting *PTBP2* exon 10 inclusion, resulting in inhibition of GBM cell proliferation and maintenance of tumour stem cells.^43^ K63‐mediated ubiquitination is a specific type of protein modification process that typically occurs at the Lys63 site of the protein.^110^ Unlike other types of ubiquitination (such as K48 ubiquitination), K63‐linked ubiquitination usually does not lead to the degradation of the modified protein but forms a persistent modification, playing a regulatory role in the cell.^111^ Notably, the latest study by Li et al. explains a new role of RBFOX2 in GBM and highlights a novel phenomenon of K63‐mediated ubiquitination. Specifically, in GBM, the upregulation of FBXO7 can dimethylate Arg341 and Arg441 of RBFOX2 through PRMT5, followed by K63‐linked ubiquitination at Lys249. This modification enhances RBFOX2 stability and upregulation. The upregulated RBFOX2 promotes the inclusion of *FoxM1* exon Va, making *FoxM1* more susceptible to MEK1 phosphorylation, thereby increasing the levels of CD44, CD9 and ID1, ultimately leading to GBM stem cell self‐renewal and mesenchymal transformation.^103^ Targeting the FBXO7‒RBFOX2 axis could become a potential strategy for GBM treatment. In malignant gliomas, RBFOX2 acts as a reader, recognising hm5C introduced by NSUN5 and TET2 on *CTNNB1* caRNA, leading to *CTNNB1* caRNA degradation. *CTNNB1* encodes the β‐catenin protein. This process inhibits the β‐catenin signalling pathway, blocking the phagocytosis of tumour‐associated macrophages and resulting in strong immune evasion in malignant gliomas.^30^

Fascinatingly, in endometrial cancer cell lines (KLE and Ishikawa), *circRAPGEF5* has been found to competitively bind to the CTD of RBFOX2, specifically residues 266−367. This interaction prevents RBFOX2 from binding to *TFRC* pre‐mRNA, showcasing the intricate regulatory network governing RNA splicing in cancer cells. This event hinders the inclusion of *TFRC* exon 4, rendering ferroptosis resistance in endometrial cancer cells.^45^

### 6.3 RBFOX2‐mediated splicing regulation in anticancer drug resistance

The emergence of drug resistance in tumour cells poses a significant obstacle to the effectiveness of chemotherapy and contributes to unfavourable prognoses.^112^ Recent studies have uncovered the pivotal role of stabilising the splicing activity of RBFOX2 in conferring resistance to anticancer therapies.^6^

TAM, a widely used selective estrogen receptor modulator, plays a critical role in BrC treatment.^113^ Notably, statistics reveal that 2 years of TAM therapy can provide long‐term therapeutic benefits lasting up to 20 years for premenopausal BrC patients with estrogen receptor‐positive (ER^+^) tumours.^114^ RBFOX2's role in interfering with the transcriptional activity of steroid receptors, including the modulation of ligands like TAM, was first reported in 2002.^11^ The RBFOX2‐DN isoform, a major negative regulator of RBFOX2's canonical splicing activity, may potentially underlie resistance to other steroid receptor modulators such as Raloxifene,^115^ Fulvestrant^116^ and Antiandrogens^117^ in ER^+^ BrC.^118^

Rebecsinib, a small molecule exhibiting the ability to counteract aberrant gene overediting prompted by malignancies, presents a hopeful avenue for addressing drug resistance and relapse in patients with high‐risk myelofibrosis and secondary AML driven by leukaemia stem cells.^119^ In cord‐blood‐derived hematopoietic stem and progenitor cells, downregulation‐induced splicing dysregulation of RBFOX2 sensitises cells to Rebecsinib treatment, as demonstrated in both colony survival and colony replating assays.^6^

## 7 TARGETING RBFOX2: UNRAVELING THERAPEUTIC STRATEGIES FOR DISEASE MODULATION

**FIGURE 5:**
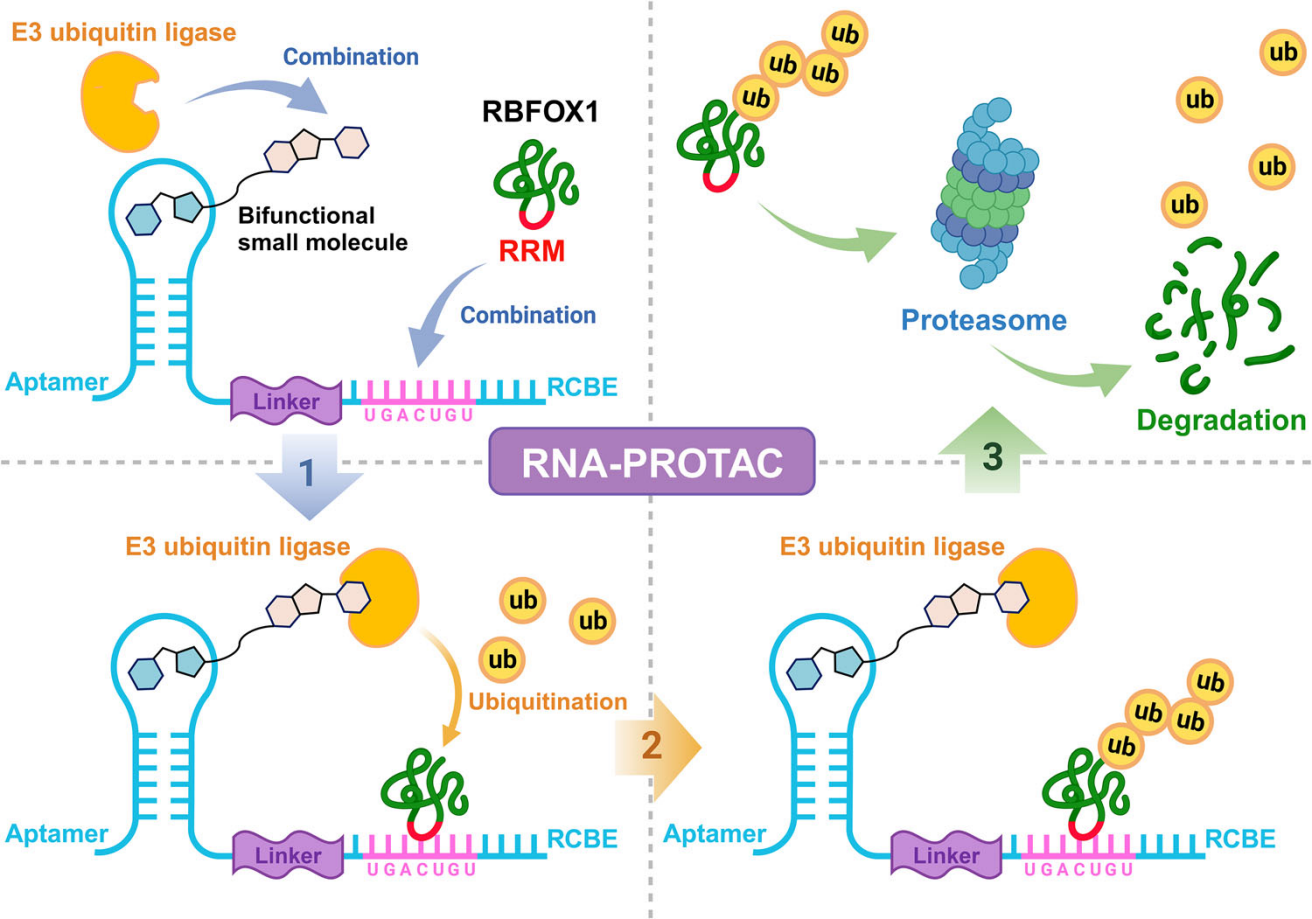
RNA‐PROTAC: targeted degradation strategy extending to RBFOX proteins. RNA‐PROTAC consists of a genetically encoded RNA scaffold (containing an aptamer, linker and a RNA consensus binding element [RCBE]) and a synthetic bifunctional small molecule (one end binds the RNA scaffold and the other end binds E3 ubiquitin ligase). When the RCBE of RNA‐PROTAC contains the RNA‐binding fox‐1 homologue 1 (RBFOX1) binding sequence (UGCAUGU), RBFOX1 can bind to the RNA scaffold of the RNA‐PROTAC. At the same time, the bifunctional small molecules on RNA‐PROTAC can recruit E3 ubiquitin ligase to the RNA scaffold in a non‐covalent manner. The recruited E3 ubiquitin ligase specifically induces the ubiquitination of the target protein (i.e., RBFOX1) and its degradation through the ubiquitin‒proteasome pathway. This targeting method is also expected to be extended to RBFOX2. PROTAC, proteolysis targeting chimera; RCBE, RNA consensus binding element; Ub, ubiquitin. Created with BioRender.com.

RBFOX2, with its multifaceted role in alternative splicing, emerges as a crucial player in the pathogenesis of CVDs, cancers and other disorders, notably contributing to treatment resistance in malignant tumours. Consequently, targeting RBFOX2 holds significant promise as a cancer therapy. Despite this potential, there is a conspicuous absence of reports on drugs specifically designed to target RBFOX2. Traditionally, RBPs have posed challenges as drug targets.^120^ However, recent advancements in protein degradation techniques, such as proteolysis‐targeting chimeras (PROTAC), have opened new avenues (Figure 5).^121^ RNA‐PROTAC, comprising a genetically encoded RNA scaffold and a synthetic bifunctional small molecule, orchestrates the degradation of target proteins through ubiquitin‐proteasome‐mediated pathways.^122^ Studies indicate that Aptamer‐based RNA‐PROTAC effectively induces the degradation of RBFOX1, a close homologue of RBFOX2, by targeting the specific RBFOX1 binding sequence (‒UGCAUGU‒).^123^ Given the structural similarity between RBFOX1 and RBFOX2 in their binding sequences (‒UGCAUG‒), Aptamer‐based RNA‐PROTAC presents a promising avenue for RBFOX2 degradation.

Additionally, targeting upstream genes that influence RBFOX2 expression provides an alternative strategy. Notably, NEK2, a cancer‐associated factor linked to tumour progression and treatment resistance, promotes the premesenchymal splicing pattern of TNBC cells by upregulating RBFOX2 expression.^7^ Despite the therapeutic potential of NEK2, no clinically approved NEK2‐targeting drugs exist. T‐1101 tosylate, a synthetic compound, induces NEK2 degradation and has shown anti‐tumour activity in HCC and BrC models.^124^ Promisingly, T‐1101 tosylate has progressed to phase I clinical trials as an orally administered anticancer agent.^124^ Moreover, imidazo pyridine derivatives, including MBM‐55 and 28e, have exhibited potent inhibitory effects on NEK2.^124^ These compounds inhibit NEK2 activity by forming conserved hydrogen bonds, effectively impeding the proliferation of diverse tumour cells, including those in BrC, HCC and CRC.^125^, ^126^

As research into RBFOX2‐related diseases deepens, the development of therapies targeting RBFOX2 becomes increasingly urgent and promising. Currently, RBFOX2‐targeted research remains exploratory, with no publicised outcomes. However, with ongoing research, it is anticipated that RBFOX2‐targeted therapies will progress to clinical applications, offering improved prognoses for patients in need.

## 8 CONCLUDING REMARKS

Alternative splicing of RNA is pivotal for modulating various isoforms of downstream genes. Among RBPs, the RBFOX family, particularly RBFOX2, plays a crucial role in alternative RNA splicing, impacting diseases such as cancer and heart disease. RBFOX2 has emerged as a diagnostic marker in cancer and a potential therapeutic target. Moving forward, RBFOX2 assumes a multifaceted role extending beyond mere disease involvement. Notably, it plays a crucial part in growth, development and maintenance of bodily homeostasis by modulating tissue‐specific alternative splicing.^127^ For instance, *Rbfox2* neural crest‐specific deletion mice, constructed using the Cre‐LoxP recombinase system, develop craniofacial malformations such as cleft palate due to dysregulation of the Rbfox2‐TGF‐β‐Tak1 signalling axis during development. This suggests that RBFOX2 expression defects are key predisposing factors for neural crest developmental defects.^128^ Moreover, in the murine liver, Rbfox2 orchestrates cholesterol homeostasis by governing the alternative splicing of *Scarb1*. These findings underscore the intricate regulatory functions of RBFOX2 in diverse physiological processes.^52^

Although RBFOX2 expression disorders have been found in various diseases, the limitations of selected experimental analysis methods and research entry points mean that most results only indicate a statistical association between RBFOX2 and disease development. The causal relationship between RBFOX2 dysregulation and disease progression cannot yet be confirmed. Currently, we can only assert that RBFOX2 disorders are key predisposing factors for HLHS and neural crest developmental defects. Future studies could involve functional experiments in the laboratory, such as gene editing technologies (meganuclease, zinc‐finger nuclease [ZFN], transcription activator‐like effector nuclease [TALEN] or CRISPR/Cas9), to artificially regulate the target gene and observe its effects on cells or model animals. Fully exploring the causal relationship between RBFOX2 dysregulation and disease development will greatly enhance our understanding of disease pathogenesis and aid in developing new treatments and preventive measures.

Current basic research has unveiled the initial understanding of how RBFOX2 regulates alternative RNA splicing. Despite this progress, the full‐length structure of RBFOX family proteins remains elusive, limiting in‐depth exploration. Future studies employing advanced techniques such as nuclear magnetic resonance or cryo‐electron microscopy are poised to reveal the complete molecular structure of RBFOX2, expanding our comprehension significantly.

Protein post‐translational modifications (PTMs), including potential phosphorylation and methylation sites within RBFOX2, are areas ripe for investigation. These PTMs likely influence RBFOX2 function and disease development. Additionally, RBFOX2 exhibits multiple isoforms regulated by alternative splicing. While existing research predominantly focuses on overall RBFOX2 expression, understanding specific isoform expression could resolve the current controversies. Future exploration of PTMs and RBFOX2 isoforms promises a comprehensive understanding of its role in cancer and other diseases.

Given its diverse roles, particularly in malignancies, targeting RBFOX2 emerges as a pivotal strategy for translating research into clinical applications. Innovative technologies such as RNA‐PROTAC and NEK2 inhibitors (such as T‐1101 tosylate, MBM‐55 and 28e) offer promising avenues for targeted degradation and reduction of RBFOX2 levels. With ongoing advancements, RBFOX2‐related research is poised to enhance diagnostic accuracy, prognostic prediction and therapeutic efficacy in the near future.

## AUTHOR CONTRIBUTIONS

*Conceptualisation, writing—original draft and visualisation*: Jinze Shen. *Writing—original draft and visualisation*: Jianqiao Shentu. *Visualisation*; Chenming Zhong and Qiankai Huang. *Conceptualisation, writing—review and editing and funding acquisition*: Shiwei Duan.

## CONFLICT OF INTEREST STATEMENT

The authors declare they have no conflicts of interest.

## ETHICS STATEMENT

Not applicable.

## Supporting information

Supporting information

## Data Availability

Not applicable.
