# Supplementary material for: RNA splicing factor RBFOX2 is a key factor in the progression of cancer and cardiomyopathy
Source: Clin Transl Med. 2024 Sep 7;14(9):e1788. doi: 10.1002/ctm2.1788 (PMC11380049; doi:10.1002/ctm2.1788)
Supplement: Supplementary file 1 — Supporting information [file CTM2-14-e1788-s001.docx]

**Supplemental Information**

Supplemental information includes one table.

**Supplementary Table 1. RBFOX2 (RBM9) exons on chromosome 22q12.3**

| **Exon** | **Chromosome** | **Start** | **Stop** |
| --- | --- | --- | --- |
| 1A | 22 | 36028824 | 36028240 |
| 1B | 22 | 35961630 | 35961563 |
| 2 | 22 | 35938897 | 35938847 |
| 1C | 22 | 35840464 | 35840192 |
| 3 | 22 | 35810004 | 35809780 |
| 4 | 22 | 35781746 | 35781600 |
| 5 | 22 | 35778075 | 35778025 |
| 6 | 22 | 35768349 | 35768257 |
| 7 | 22 | 35765483 | 35765423 |
| 8 | 22 | 35761468 | 35761415 |
| 9 | 22 | 35761294 | 35761202 |
| 10 | 22 | 35760020 | 35759888 |
| 11A | 22 | 35756144 | 35756105 |
| 11B | 22 | 35750457 | 35750426 |
| 12 | 22 | 35746561 | 35746473 |
| 13 | 22 | 35745995 | 35745923 |
| 14 | 22 | 35744249 | 35738736 |

Genomic coordinates of human exons utilized to construct the gene structure depicted in Figure 1 are provided herein. The numbering corresponds to the exon sequence illustrated in the figure, diverging from the previously employed numbering system. These coordinates are referenced according to hg38 and are accessible via the National Center for Biotechnology Information (NCBI) at https://www.ncbi.nlm.nih.gov.
